# Erysipelothrix rhusiopathiae-associated bloodstream infection in a patient with systemic lupus erythematosus: a case report and literature review

**DOI:** 10.1099/acmi.0.000881.v3

**Published:** 2024-11-06

**Authors:** Calvin Ka-Fung Lo, Cole Schonhofer, Neil Mina, Shazia Masud, Patrick Ho Pun Wong, Michael G. Chapman

**Affiliations:** 1 Department of Pathology and Laboratory Medicine, University of British Columbia, Vancouver, British Columbia, Canada; 2 Division of Medical Microbiology, Surrey Memorial Hospital, Fraser Health, Surrey, British Columbia, Canada; 3 Division of Infectious Diseases, Surrey Memorial Hospital, Fraser Health, Surrey, British Columbia, Canada

**Keywords:** bloodstream infection, *Erysipelothrix*, systemic lupus erythematosus, zoonotic

## Abstract

**Introduction.** Systemic human infections caused by *Erysipelothrix rhusiopathiae* have been increasingly reported especially within immunocompromised hosts and those with significant occupational exposure to livestock and aquatic animals. We report a case of *E. rhusiopathiae* bacteraemia in a patient with systemic lupus erythematosus (SLE) and present a literature review on clinical outcomes and microbiologic diagnosis for this organism.

**Casepresentation.** A 43-year-old female patient was reporting a 1-month history of intermittent fevers. She recently increased her immunosuppression medication for her underlying SLE on the advice of her rheumatologist. The patient sustained a finger laceration from butchering cattle meat 2 weeks after the onset of her initial symptoms, with worsening index finger swelling and increased febrile episodes. Two weeks post-injury, multiple blood cultures were drawn, and each isolated Gram-positive bacilli. Given her recurrent intermittent fevers, there was a concern for ongoing infection, and therefore, intravenous vancomycin was started with prompt referral to an outpatient parenteral antibiotic therapy clinic. The Gram-positive bacillus was confirmed as *E. rhusiopathiae* via matrix-assisted laser desorption/ionization-time of flight analysis. Given intrinsic resistance to vancomycin, vancomycin was switched to intravenous ceftriaxone as targeted antimicrobial therapy for 2 weeks. Reassuringly, there was no echocardiographic evidence of infective endocarditis, warranting the prolonged treatment course. Post-treatment, she remained symptom-free with the resolution of joint symptoms and fevers.

**Conclusion.** Our report illustrates a case of *E. rhusiopathiae* bacteraemia from an immunodeficient host, with prompt microbiologic diagnosis and intervention with appropriate antimicrobial coverage. Literature reflects the rarity of this infection, predilections to specific susceptible hosts and the importance of raising awareness of zoonotic infections.

## Data Summary

All data generated or analysed during this study are included in this published article (and its supplementary information files). Please see Tables 1, 2 and 3 for data extracted from eligible articles within our literature review.

## Introduction

*Erysipelothrix rhusiopathiae* is a Gram-positive rod-shaped bacterium (bacillus) distributed worldwide. They have been isolated from soil, food scraps and water contaminated by infected animals and can survive for months in soil. This bacterium was first isolated by Robert Koch in 1878 and eventually identified as the causative agent for erysipelas in pigs in 1886 by Löffler [1]. The organism was first established as a human pathogen in 1909 when Rosenbach isolated it from a patient presenting with localized cutaneous lesions [1]. Of note, the most significant commercial impact would be disease within its major reservoirs (i.e. swine); however, the infection also extends to poultry and sheep [1].

Given predilection of *E. rhusiopathiae* circulating around its animal reservoirs, human infection is considered a zoonosis and remains closely linked to occupational exposure with prolonged animal contact or inoculation with infected animals [1,2]. Reported risk factors for *Erysipelothrix*-associated infections primarily include occupations involving close contact with animals or animal products. This includes fishing/seafood industry, swine and cattle farmers and butchers and veterinarians. There have been rare reports of acquiring infection via ingestion of undercooked pork and dog or cat bites [3]. There has been an increasing report of systemic *Erysipelothrix*-associated infections without infective endocarditis, including less commonly reported complications, such as osteomyelitis, septic arthritis, peritonitis, abscesses and meningitis [4]. A key study by Tan *et al.* in 2017 involved a 22-year retrospective review between 1994 and 2016 at the Mayo Clinic, Rochester, Minnesota [5]. In addition to the zoonotic exposure, predisposing factors for systemic infection included iatrogenic or disease-associated immunosuppression (e.g. solid organ or haematopoietic stem cell transplant), chronic alcohol consumption, diabetes mellitus and chronic kidney disease.

Given its similar morphology based on Gram stain and microbiologic characteristics (e.g. catalase test), it can potentially be confused with other Gram-positive bacilli such as *Listeria monocytogenes* and *Arcanobacterium haemolyticum*; however, automated identification systems and matrix-assisted laser desorption/time-of-flight (MALDI-TOF) mass spectroscopy has improved diagnosis of this pathogen [2].

We report here a case of *E. rhusiopathiae* bacteraemia in a patient with systemic lupus erythematosus (SLE) on immunosuppressants whose blood culture results were initially dismissed as contaminant. We also performed a literature review to summarize human infections caused by *E. rhusiopathiae* with a focus on immunosuppressed hosts, to review risk factors, management and clinical outcomes.

## Case presentation

A 43-year-old female with SLE presented in mid-October 2023 with a 4-week history of recurrent fevers up to 38.5 °C every 4 to 5 days. Of note, she recently immigrated from Nigeria to Canada ~1 year prior to her clinical presentation. For her SLE (diagnosed in 2019), she was receiving prednisone 10 mg PO daily and hydroxychloroquine 100 mg *per os* (by mouth) (PO) *bis in die* (twice daily) (BID), with full adherence and no missed doses. She does not have outpatient rheumatology follow-up and corresponds infrequently with her rheumatologist in Nigeria whenever symptomatic.

One month before presentation (mid-September 2023), she reported new onset of fevers (peak 38.5 °C) with spontaneous resolution. There was neither significant travel history nor recent sick contact exposures. She denied having any pets at home, recent animal or insect bites. Specifically, she denied contact with pigs or livestock, handling of fish and aquatic animals. Through telehealth with her rheumatologist in Nigeria, she was advised to increase medication doses on 25 September 2023 for presumed lupus flare (i.e. prednisone increased to 30 mg PO daily; hydroxychloroquine increased to 200 mg PO BID).

Within 2 weeks after her SLE medication, doses were increased (4 October), and she experienced intermittent chest tightness, fevers (peak 38.3 °C), chills and headaches. She did not report any dyspnoea, cough, gastrointestinal or urinary symptoms. One week later, she noted recurrent pleuritic chest pain, fevers (similar peak as mentioned earlier) and myalgias and hence rushed to her community hospital emergency department (ED) for urgent assessment on 14 October 2023. Although her electrocardiogram (ECG), chest X-ray and troponin levels were unremarkable, she had elevated peripheral white cell count (17.4; normal range 4.5–11.0×10^9^ l^−1^), neutrophils (15.1; normal range 2.0–8.0×10^9^ l^−1^) and C-reactive protein (CRP) (67.3; normal <3.1 mg l^−1^). Her syndrome was provisionally diagnosed as a lupus flare (the emergency note briefly commented about potential viral infection although they said no localizing symptoms besides intermittent chest pain and pericarditis/myocarditis being ruled out with normal ECG and troponin), and she was discharged home with increased prednisone dosage (to 50 mg PO daily for 1 week, before progressive tapering at 10 mg per week). Due to persistent low-grade fevers between 38.0 and 38.5 °C, she returned to the hospital the following day with white cell count increased to 25.2, neutrophils to 23.6 and CRP to 79.3. Underlying infection became more of a clinical concern when peripheral blood cultures grew 2 of 2 sets of Gram-positive bacilli. Intravenous vancomycin 1000 mg q12h was initiated and urgently referred to the outpatient infectious diseases clinic for our assessment. There were no reported headaches, sore throat, cough, abdominal pain, vomiting, diarrhoea, rashes or joint pain.

At our infectious diseases clinic assessment on 16 October 2023, we further inquired about exposure risks, and she revealed about participating in a group activity in late September involving butchering freshly slaughtered cattle beef from a local farm in British Columbia. While dissecting beef from bone-in portion, she accidentally inoculated her left index finger (laceration near her proximal interphalangeal joint) with the blood-contaminated knife. This incident in late September occurred ~4–5 days after she had her medication dosages increased.

Subsequently, she developed significant swelling around her metacarpalphalangeal joint and proximal segment of her left index finger within 48 h post-injury (3 October). She described it as throbbing pain with pruritic sensation, a dark ‘bruising hue’ around her inoculation site and difficulty with finger flexion. This joint swelling coincided with her recurrence of fevers described in early October. Interestingly, her left index finger swelling already self-resolved by 15 October prior to antibiotic administration.

On our physical examination, she was alert and oriented and had stable hemodynamics with blood pressure of 140/71, pulse rate of 85 and oral temperature of 36.9 °C (afebrile on our assessment). Lung auscultation was normal without any bilateral adventitious sounds, and cardiovascular examination did not reveal any murmurs or other stigmata of infective endocarditis. She had no hepatosplenomegaly and no tenderness palpating her abdomen including the right upper quadrant, suprapubic or costovertebral angle region. Detailed examination did not reveal any additional violaceous or cutaneous lesions elsewhere on the body, and there was no evidence of lymphangitis on upper extremities. A 1.5–2.0 cm scabbed lesion was noted over the lateral aspect of her left index finger in keeping with her inoculation injury, and there was no evidence of erythema, purulent discharge or eschar. She had symmetrical motion of her fingers and wrists.

**Fig. 1.**
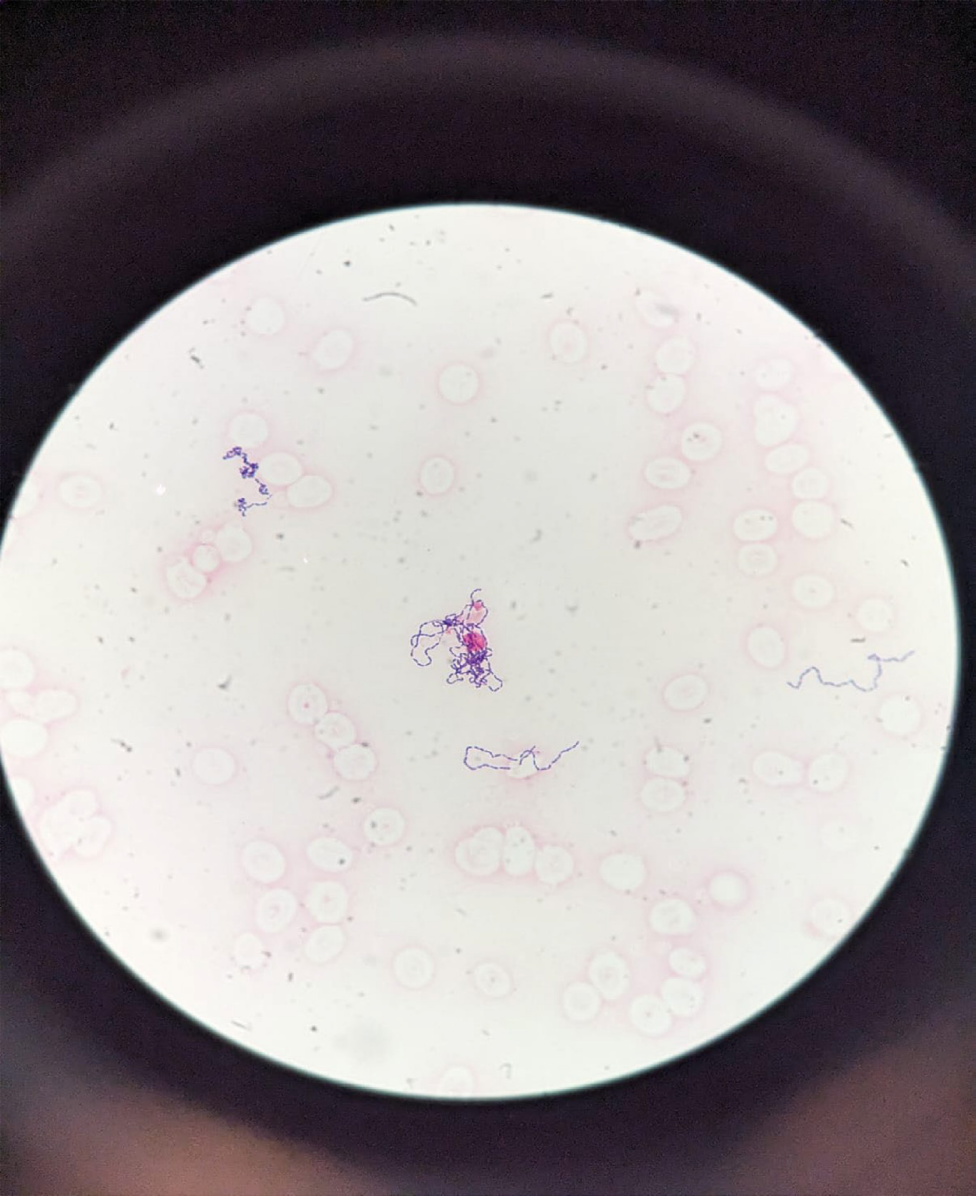
Gram stain of blood culture sample. Demonstration of Gram-positive bacilli with non-branching filamentous morphology under light microscopy, 100×.

Review of her chest X-ray imaging taken during ED presentation was negative for any intrathoracic pathology. The nasopharyngeal swab was negative for influenza A and B and SARS-CoV-2. Peripheral blood cultures were positive for 2 of 2 sets of Gram-positive bacilli identified as *E. rhusiopathiae* (susceptibilities pending at time of assessment, Fig. 1).

Initially, the Gram-positive bacilli had beige, non-haemolytic colonies that were flattened and rough in appearance, catalase-negative. It was queried as a potential contaminant; however, further growth occurred in multiple blood culture bottles, and her recently increased immunosuppression were concerning for an underlying persistent bloodstream infection with potential dissemination risk. Our suspicion was confirmed with definitive identification of *E. rhusiopathiae* using MALDI-TOF VITEK MS V3 (Bruker, Billerica, MA, USA). MALDI-TOF run report and scatter gram of the isolate (tested in duplicate) from this case are included as Supplementary File B (available in the online Supplementary Material). Given recurrent fevers occurring in temporal relation to her inoculation injury from cattle blood exposure, this was attributed as the causative pathogen. Subsequent repeat peripheral blood cultures collected 24 h later continued to isolate the same organism.

**Fig. 2.**
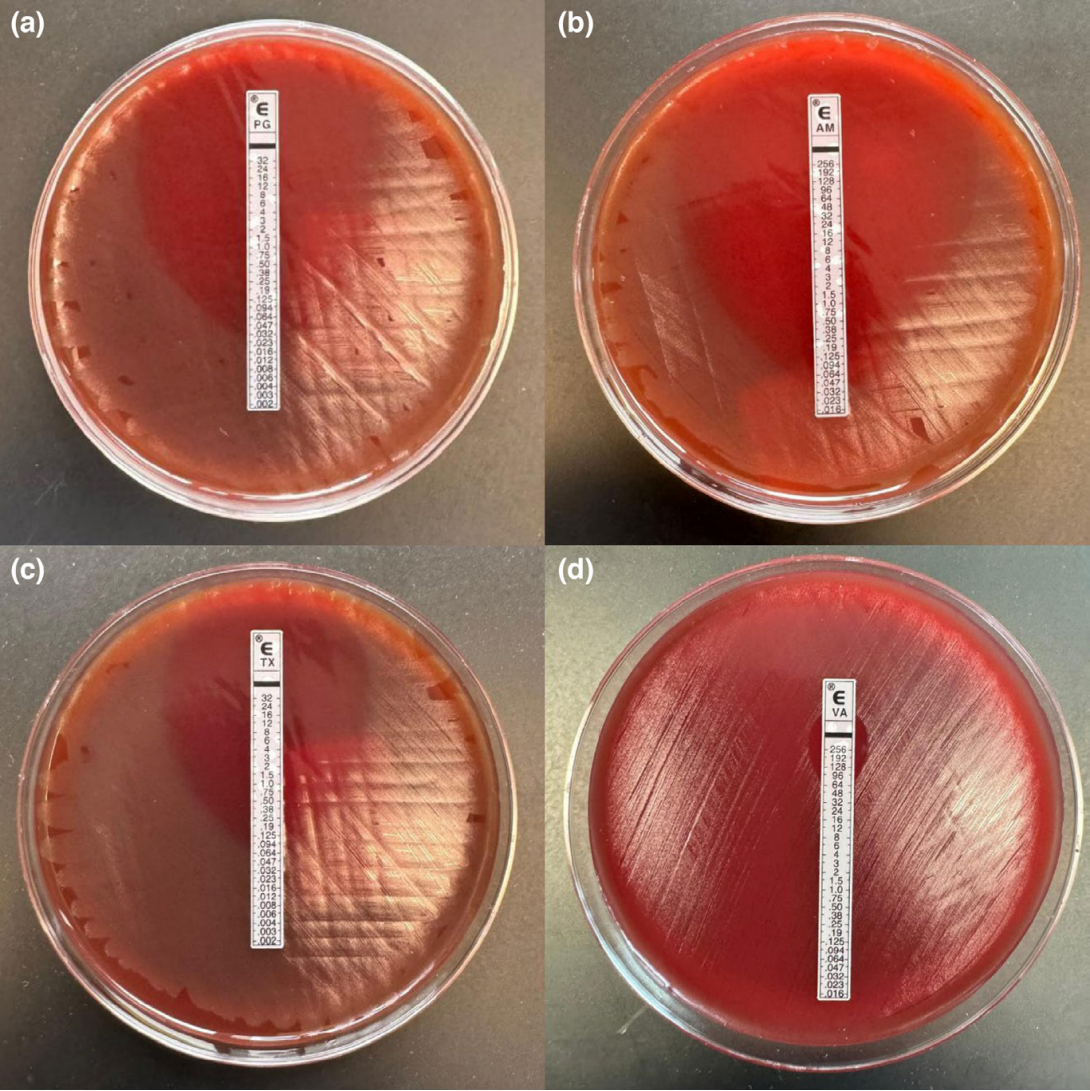
Antimicrobial susceptibility results for our *E. rhusiopathiae* isolate, via E-test strip gradient diffusion. E-tests for the corresponding antibiotics: (a) penicillin G, (**b**) ampicillin, (**c**) ceftriaxone and (**d**) vancomycin. As per CLSI M45 third edition (2016) [6], the isolate was susceptible to penicillin G (MIC 0.023 µg ml^−1^, susceptible if ≤0.12 µg ml^−1^), ampicillin (MIC 0.047 µg ml^−1^, susceptible if ≤0.12 µg ml^−1^) and ceftriaxone (MIC 0.064 µg ml^−1^, susceptible if ≤1 µg ml^−1^). It was resistant to vancomycin (MIC 64 µg ml^−1^, with no breakpoint given *Erysipelothrix* is intrinsically resistant).

Inoculum was prepared from blood agar after incubating isolate for 24 h at 35 °C (± 2°), with suspension made to 0.5 McFarland standard. The MICs were determined using *E*-test gradient strip (bioMérieux, France) as part of susceptibility testing for this isolate (Fig. 2). Findings were interpreted as per Clinical and Laboratory Standards Institute (CLSI) M45 (Methods for Antimicrobial Dilution and Disk Susceptibility Testing of Infrequently Isolated or Fastidious Bacteria, Edition 3) [6]. Susceptibility results showed susceptibility to penicillin, ampicillin and ceftriaxone and resistance to vancomycin (Fig. 2). Hence, vancomycin was changed to intravenous ceftriaxone 2 g daily as of 16 October 2023. Subsequent blood cultures were negative after ceftriaxone was initiated for ~48 h.

## Diagnosis, treatment and follow-up

Although she had no evidence of septic arthritis at the time of our assessment, her unclear duration of bloodstream infection and delay in antimicrobial initiation post-injury were concerns for developing complicated bacteraemia. Reassuringly, her transthoracic echocardiogram was absent for hemodynamically significant valvular dysfunction, vegetation or perivalvular abscesses suggestive of infective endocarditis. The patient was clinically stable and thus discharged to complete her antimicrobial course via our home intravenous antibiotic therapy programme for 14 days total duration (completed on 30 October 2023). She tolerated her ceftriaxone well, defervesced within 72 h including blood culture clearance, and had resolution of her finger lesion. The patient remained symptom-free 3 months post-completion of therapy, and we arranged outpatient rheumatology follow-up locally around her community for ongoing SLE management.

## Discussion

Gram stain interpretation can be challenging, especially its variable, pleomorphic morphology ranging from coccoid to long, filamentous forms; the presence of both morphotypes can confuse clinicians to think of polymicrobial presence or possibly confuse as normal skin microbiota such as *Corynebacterium* [2]. Hence, in the setting of immunocompromised patients, the presence of pleomorphic Gram-positive bacilli isolated from blood culture bottles should not be routinely dismissed as contaminants, especially if linked to occupational exposure to animal tissues or products. The number of recognized infections due to *Erysipelothrix* spp. may rise in the future because of improved awareness surrounding the variable characteristics of this pathogen, availability of improved laboratory identification databases and increasing overlapping interactions between agricultural animals and humans. Accurate identification of Gram-positive bacilli such as *Erysipelothrix* spp. from clinical cultures with suspected infections will expedite appropriate targeted therapy.

**Fig. 3.**
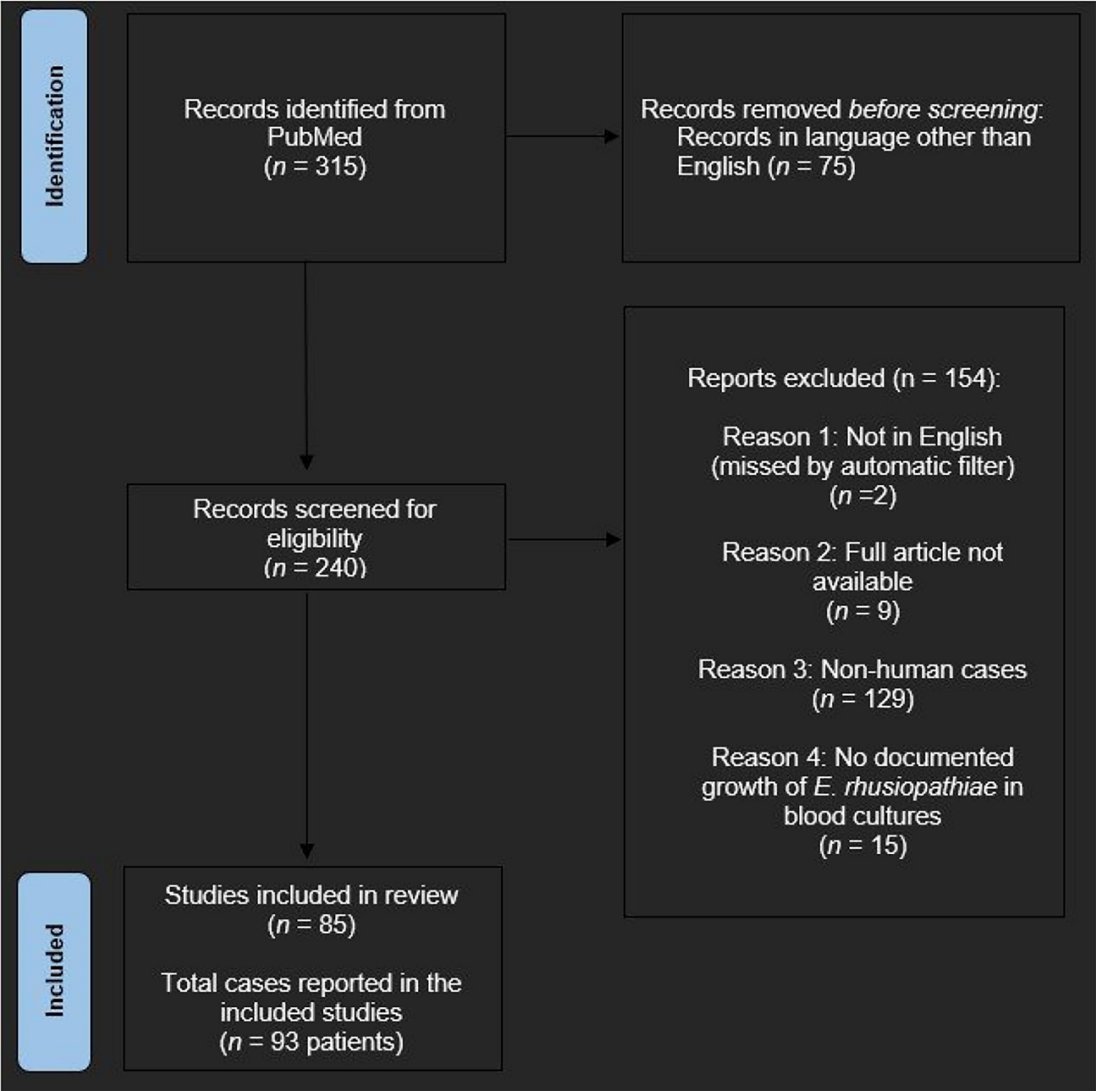
Flowchart of literature review criteria from PubMed database. Search was done on PubMed via: ‘Erysipelothrix AND (bacteremia OR septicemia OR endocarditis OR immunocompromise)’.

A comprehensive literature review was conducted using the PubMed database to identify cases of *Erysipelothrix* spp. bacteraemia in both immunocompetent and immunosuppressed patients (Fig. 3). Search phrase used was ‘*Erysipelothrix* AND (bacteremia OR septicemia OR endocarditis OR immunocompromise)’. Inclusion criteria included English language and human infections only, availability of the full article with primary patient data and confirmed *E. rhusiopathiae* bacteraemia via blood culture. Overall, we found 315 articles from 1950 to June 2024, of which 85 articles detailing 93 cases met the inclusion criteria (summarized in Table 1) [5,7,91]. The identical search strategy was repeated on MEDLINE-OVID but did not yield any additional cases.

**Table 1.** Literature review on reported human cases of *E. rhusiopathiae* bacteraemia (PubMed database)

Patient characteristics and variables	Pooled results (*n*=93 cases)
Age in years, median (range; IQR)	54 (neonate to 91; 47–62)
Sex	Male (64, 68.8%)
	Female (29, 31.2%)
Comorbidities	Alcohol use (23, 24.7%)
	Hypertension (14, 15.1%)
	Autoimmune disease (15, 16.1%)
	Type 2 diabetes mellitus (9, 9.7%)
	Structural heart disease (8, 8.6%)
	Coronary artery disease (6, 6.5%)
	Malignancy (5, 5.4%)
	COPD/asthma (5, 5.4%)
	Liver disease (7, 7.5%)
	Chronic kidney disease (8, 8.6%)
	Dyslipidaemia (6, 6.5%)
	None (13, 14.0%)
Immunosuppressed*	22 (23.7%)
Endocarditis	Present (52, 55.9%)
	Absent (41, 44.1%)
Superficial infection and/or superficial injury	Present (37, 39.8%)
	Absent (56, 60.2%)
Known or suspected exposure**†**	Present (76, 81.7%)
	Absent (17, 18.3%)
Antibiotics used for treatment after ID**‡**	Penicillin (45, 48.4%)
	Ceftriaxone (20, 21.5%)
	Ampicillin/amoxicillin (11, 10.8%)
	Amoxicillin–clavulanate (3, 3.2%)
	Other cephalosporins (10, 11.8%)
	Fluoroquinolones (5, 5.4%)
	Clindamycin (4, 4.3%)
	Macrolides (4, 4.3%)
	Carbapenems (2, 2.1%)
Antibiotic duration**§**	At least 6 weeks (35, 37.6%)
	>4 weeks, <6 weeks (29, 31.2%)
	<4 weeks (14, 15.1%)
	No antibiotics (1, 1.1%)
	Duration not stated (14, 15.1%)
Identification method[Table-fn T1_FN5]||	Culture and biochemical tests alone (67, 72.0%)
	Vitek-II ID card (7, 7.5%)
	MALDI-TOF (8, 8.6%)
	API-Coryne ID strip (7, 7.5%)
	BD Phoenix (2, 2.2%)
	Biofire Blood Culture ID panel (1, 1.1%)
	16S RNA sequencing (4, 4.3%)

* Further detailed in Table 2.

† Exposures defined as: direct contact with animals or animal products and/or occupational risks such as farming, fishing, and hunting. Patients were considered ‘exposed’ if the study authors noted a suspected or confirmed exposure event or activity likely to be attributive of an exposure source for *Erysipelothrix*.

‡ Included all antibiotics used after the identification of *E. rhusiopathiae* bacteraemia. Patients were often treated with multiple antibiotics, either in combination therapy, if clinically indicated, or oral transition.

§ Maximum treatment duration identified in this review was 12 weeks.

|| Three studies used multiple identification modalities. One study used both Vitek and MALDI-TOF, one study used both MALDI-TOF and 16sS -RNA sequencing, and one study used API-Coryne and 16S- RNA.

COPDchronic obstructive pulmonary disease

**Table 2.** Characteristics of immunosuppressed patients with *E. rhusiopathiae* bacteraemia (PubMed database)

Immunosuppressed patient characteristics	Pooled results (*n*=22)
Median age in years (range; IQR)	53 (18–72; 43.0–58.0)
Sex	Male (13, 59.1%)
	Female (9, 40.9%)
Immune suppression cause	Treatment for underlying disease (19 cases)
	Haematologic malignancy (3 cases)
Haematologic malignancy(*n*=3)	Chronic lymphocytic leukaemia (2, 66.6%)
	Myeloma (1, 33.3%)
Underlying disease treated with immunosuppressing medications*(*n*=19)	SLE (7, 36.8%)
	Crohn’s disease (2, 10.5%)
	Chronic lymphocytic lymphoma (1)
	Rheumatoid arthritis (1)
	Psoriatic arthritis (1)
	Giant cell arteritis (1)
	Ankylosing spondylitis (1)
	Renal transplant (1)
	Myocarditis (1)
	Nephrotic syndrome (1)
	Hypopituitary requiring steroid replacement (1)
	Paroxysmal nocturnal haematuria (1)
Immunosuppressing medications*(*n*=19)	Steroid only (9, 47.4%)
	Steroid + adjunct^†^ (7, 36.8%)
	Biologic (3, 15.8%)
Endocarditis	Present (7, 31.8%)
	Absent (15, 68.2%)
Known or suspected exposure	Present (16, 72.7%)
	Absent (6, 27.3%)
Superficial infection and/or likely portal of entry (wound, cut, etc.)	Present (14, 63.6%)
	Absent (8, 36.4%)
Antibiotic duration	At least 6 weeks (5, 22.7%)
	4 to <6 weeks (9, 40.9%)
	<4 weeks (6, 27.3%)
	Duration not stated (2, 9.1%)

* *n*=19 for the 19 patients taking immunosuppressing medications.

† aAdjunct medications included Aazathioprine, Hhydroxychloroquine, Ccladribine, mMethotrexate, cCyclosporine, and Ccyclophosphamide.

The median patient age was 54 years, ranging from neonatal to 91 years old, and the majority were male (68.8%). With the exception of one neonatal case [42], the remainder were adult patients. The vast majority resolved fully with antibiotic treatment and nine cases (9.7%) ended in death. Most patients (86.0%) had underlying comorbidities as summarized in Table 1, although 13 (14.0%) were otherwise healthy or did not have comorbidities listed. The most common comorbidities included alcohol use (23 cases, 24.7%), hypertension (14 cases, 15.1%), autoimmune disorders (15 cases, 16.1%) and diabetes (9 cases, 9.7%). Twenty-two cases involved immunosuppressed patients (23.7%). Seventy-six cases (81.7%) had known or suspected exposures consistent with *Erysipelothrix* infection, such as through farming, fishing, hunting or other contact with animals and/or handling of raw animal products. Thirty-seven (39.8%) had a likely entry portal for bacteraemia on history or presentation such as cutaneous infections or wounds.

Fifty-two patients (55.9%) had confirmed endocarditis via echocardiograph or clinical criteria, while there were 41 cases of non-endocarditis-related bacteraemia (44.1%). In terms of treatment regimens, 35 patients received ≥6 weeks of antibiotic treatment (37.6%), 29 received between 4 and 6 weeks (31.2%), while 14 received <4 weeks (15.1%). Of those 14 with short duration, 13 were cases of non-endocarditis-related bacteraemia. Fourteen of 93 cases did not state treatment duration (15.1%), while 1 patient clinically resolved without any antibiotic treatment. The most common antibiotics used in treatment regimens were penicillin (45, 48.4%) and ceftriaxone (20, 21.5%). Other antibiotics used included amoxicillin or ampicillin (11, 11.8%), other cephalosporins (10, 10.8%), fluoroquinolones (5, 5.4%), macrolides (4, 4.3%), clindamycin (4, 4.3%) and carbapenems (2, 2.1%).

Twenty-two cases involved directly immunosuppressed patients, either iatrogenically (e.g. steroids, biologics or chemotoxic agents) or intrinsically (e.g. haematologic malignancy), as shown in Table 2 [5,10, 15, 16, 22, 24, 25, 30, 33, 38, 40, 41, 43, 54, 57, 61, 74, 77, 82, 84]. Median age of immunosuppressed patients was similar at 53 years. Of the 18 patients taking immunosuppressing medications, 9 were on steroid monotherapy, 7 were on steroid therapy along with other immunosuppressants and 3 were on biologic therapy. The underlying reasons for immunosuppression with medication are listed in Table 2. Interestingly, SLE was the most common reason (7/19 cases, 36.8%) for immunosuppression found amidst our search. Of the immunosuppressed patients, seven developed endocarditis (31.8%), suggesting that immunocompromised patients may be more at risk for non-endocarditis-related bacteraemia with *Erysipelothrix*. This has been noted previously as well [33]. Of the 15 who did not develop endocarditis, other significant complications included septic shock [61], pancreatic collection [30], tenosynovitis [38], meningitis [43] and pneumonia [77], as well as several superficial infections such as cellulitis. All 22 patients had positive outcomes with antibiotic treatment with no deaths. Sixteen of these patients had known or suspected exposures (72.7%), which is slightly less than the proportion in the larger cohort (76/93, 81.7%), and 14 patients had preceding or concurrent superficial infections, and/or a likely entry wound on history (63.6%), which is higher than the proportion in the overall cohort (37/93, 39.8%). As was also the case with our reported patient, it appears that immunocompromised patients may be more susceptible to superficial *Erysipelothrix* infections transitioning to bacteraemia.

Our patient presented here has been on immunosuppressing medications for SLE for at least 4 years pre-presentation. As detailed earlier, we identified seven other cases of *Erysipelothrix* bacteraemia in patients with underlying SLE, as summarized in Table 3. The median age of these patients was 43 years, and all seven were females. Each of these patients was on immunosuppressing medications, with three on steroid monotherapy and four on steroids plus adjuncts such as mycophenolate, azathioprine, hydroxychloroquine, cyclophosphamide and cyclosporine. Four of the seven patients had no identified exposures to *Erysipelothrix* infection. None were noted to misuse alcohol, the most common risk factor in the overall group. Three of these seven were found to have endocarditis (42.8%), which is again lower than the overall rate we found in other reported cases. There have also been reports of other *E. rhusiopathiae* infections such as septic arthritis in SLE patients, but these were not included in our search due to the lack of positive blood cultures [92,93]. It appears that SLE patients are less likely to present with the classic risk factors or exposures associated with *Erysipelothrix* infection.

**Table 3.** Summary of *E. rhusiopathiae* bacteraemia in patients with systemic lupus erythematosus (PubMed database, *n*=7)

Reference no.	Age, sex	Immunosuppressing medication	Known exposure	Clinical course	Other infection sites	Time from symptom onset to bacteraemia diagnosis (days)	Antibiotics	Hospitalization (days)
[33]	18, F	Glucocorticoids	Occasional animal contact	3 weeks fever, arthralgia, erythematous, migratory and nonpruritic rash	Diffuse cutaneous	21	Penicillin IV 2 weeks -> amoxicillin PO 1 week	na
[84]	43, F	Prednisolone	No	1 week × erythematous rash with bullae; treated as SLE flare with steroid; BC and WC grew ER; diagnosed as ER septicaemia with SLE flare	Diffuse cutaneous	14	Cefazolin IV × 2 weeks -> PO amoxiciilin × 2 weeks	32
[82]	29, F	Prednisolone and cyclophosphamide	No	Fever, arthralgia, heart murmur; diagnosed as aortic endocarditis via echo	Aortic valve endocarditis	Not stated	IV penicillin × 20 days -> IV cefazolin 10 days	32
[22]	31, F	Mycophenolate	Cut hand while handling fish	1 month of fever unresponsive to two short courses of antibiotics; echo diagnosis as tricuspid endocarditis	Tricuspid valve endocarditis	30	Ceftriaxone IV -> PO amoxicillin total 6 weeks	10
[5]	58, F	Azathioprine, hydroxychloroquine and prednisone	Cut finger while handling fish/chicken	One day prior, cut finger while slicing fish/chicken; cellulitis with sepsis	Localized cellulitis	1	Ceftriaxone × 2 weeks -> ciprofloxacin PO × 2 weeks	7
[30]	45, F	Cyclosporine and methylprednisolone	No	Four febrile episodes × 2 weeks; antibiotics started 4 days post-admission; pancreatic collection noted on CT	Pancreatic collection	22	IV levofloxacin × 1 week -> 3 weeks PO	11
[43]	56, F	Prednisolone	None	ITP treated with prednisolone × 1 month, then fever and altered mental status (query meningitis) BC+ for ER, endocarditis confirmed on echo; SLE diagnosed during admission; mitral valve replacement (day 10 of admission)	Mitral valve endocarditis with query CNS septic emboli	1	Ceftriaxone (duration not stated)	32

BCblood cultureCNScentral nervous systemER*Erysipelothrix rhusiopathiae*ITPimmune thrombocytopenic purpuraWCwound culture

## Identification and antimicrobial susceptibility testing of *Erysipelothrix* spp.

In most cases, *Erysipelothrix* was either identified solely by culture and biochemical properties, or additional modalities were not stated (67 cases, 72.0%). Classically, *E. rhusiopathiae* can be distinguished from other Gram-positive bacilli based on morphological and biochemical tests including non-motility, hydrogen sulphide production and negative oxidase and catalase activity. Alternative modalities such as MALDI-TOF (8 cases, 8.6%), Vitek-II ID cards (7 cases, 7.5%), API-Coryne ID strips (7 cases, 7.5%), BD Phoenix Panel (2 cases, 2.2%), Biofire Blood culture ID panel (1 case, 1.1%) were employed less frequently. Confirmatory identification with 16S RNA sequencing was pursued in four cases (4.3%).

There are currently no guidelines for clinical management of *Erysipelothrix* infections; however, CLSI M45 provides interpretive breakpoints for broth microdilution testing with penicillin, ampicillin, cefepime, cefotaxime, ceftriaxone, imipenem, meropenem, erythromycin, ciprofloxacin, gatifloxacin, levofloxacin and clindamycin [6]. The European Committee on Antimicrobial Susceptibilities breakpoints are not available for this organism. Resistance to clindamycin, erythromycin and fluoroquinolones has been reported, and susceptibility testing is warranted if these drugs are considered for treatment. There have been reports of infections with penicillin-resistant strains as well, although they remained cephalosporin and carbapenem susceptible [51,67]. CLSI breakpoints for resistance exist only for erythromycin and clindamycin, and non-susceptible results for other drugs should be investigated and confirmed.

## supplementary material

10.1099/acmi.0.000881.v3 Uncited Supplementary Material 1.

10.1099/acmi.0.000881.v3 Uncited Supplementary Material 2.

10.1099/acmi.0.000881.v3 Uncited Supplementary Material 3.

## Abbreviations

BID: *bis in die* (twice daily)
CLSI: Clinical and Laboratory Standards Institute
CRP: C-reactive protein
ECG: electrocardiogram
ED: emergency department
EUCAST: European Committee on Animicrobial Susceptibilities
IQR: interquartile range
IV: intravenous
MALDI-TOF: matrix-assisted laser desorption/time-of-flight
PO: *per os* (by mouth)
RNA: ribonucleic acid
SLE: systemic lupus erythematosus
