# Supplementary material for: Erysipelothrix rhusiopathiae-associated bloodstream infection in a patient with systemic lupus erythematosus: a case report and literature review
Source: Access Microbiol. 2024 Nov 6;6(11):000881.v3. doi: 10.1099/acmi.0.000881.v3 (PMC11649197; doi:10.1099/acmi.0.000881.v3)
Supplement: Uncited Supplementary Material 2. [file acmi-6-00881-s002.pdf]

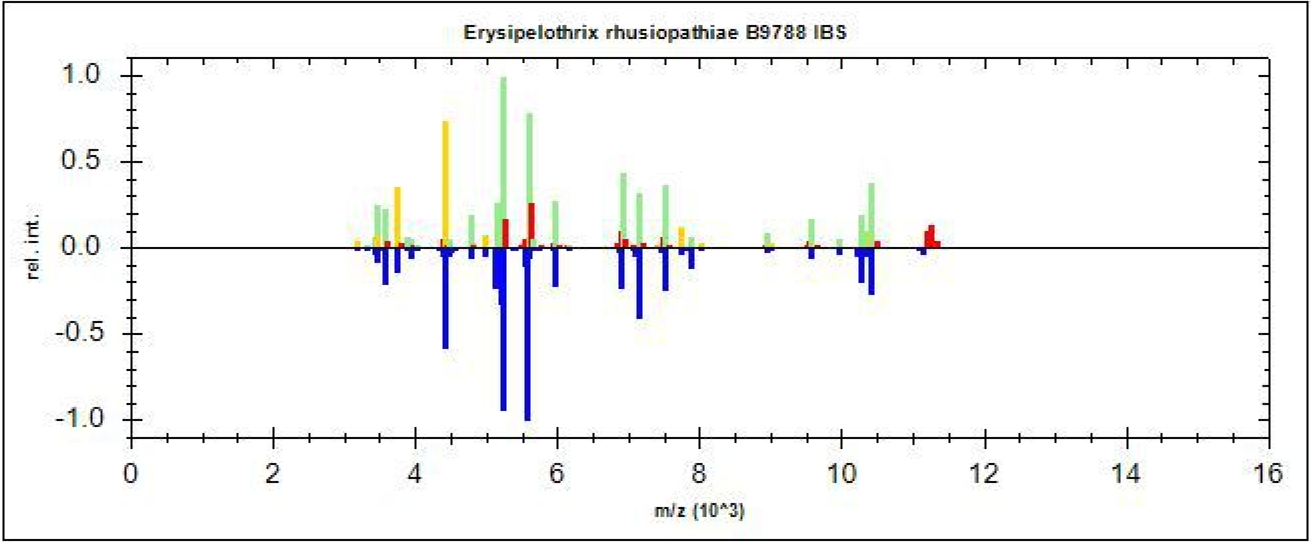
Erysipelothrix rhusiopathiae B9788 IBS
1.0
0.5
0.0
-0.5
-1.0
rel. int.
0
2
4
6
8
10
12
14
16
m/z (10^3)

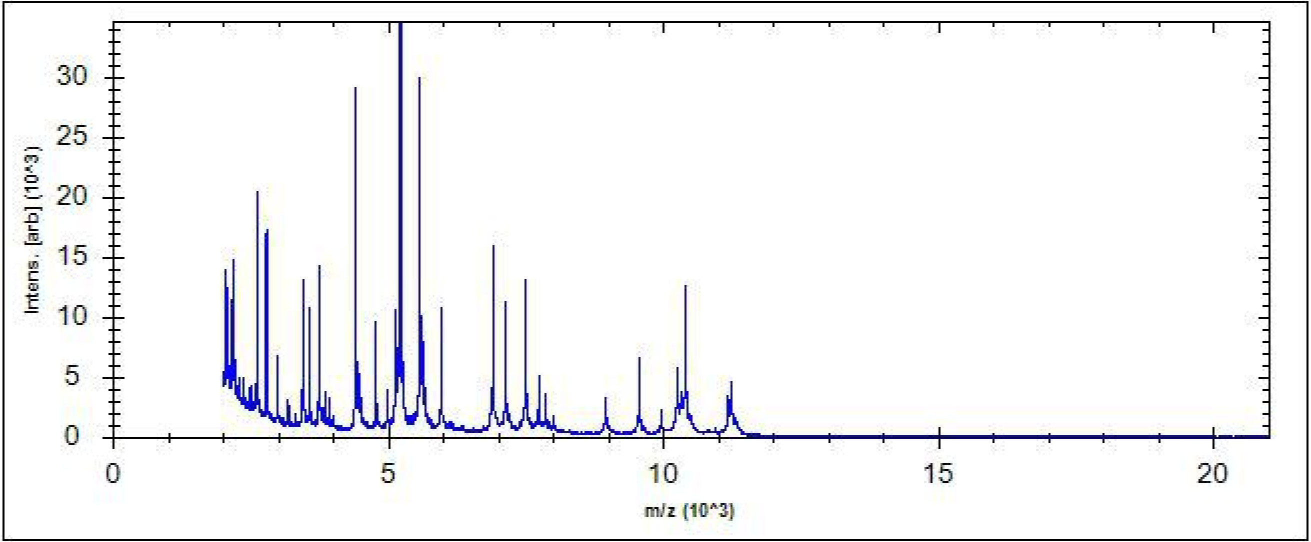
Intens. [arb] (10^3)
30
25
20
15
10
5
0
0
5
10
15
20
m/z (10^3)

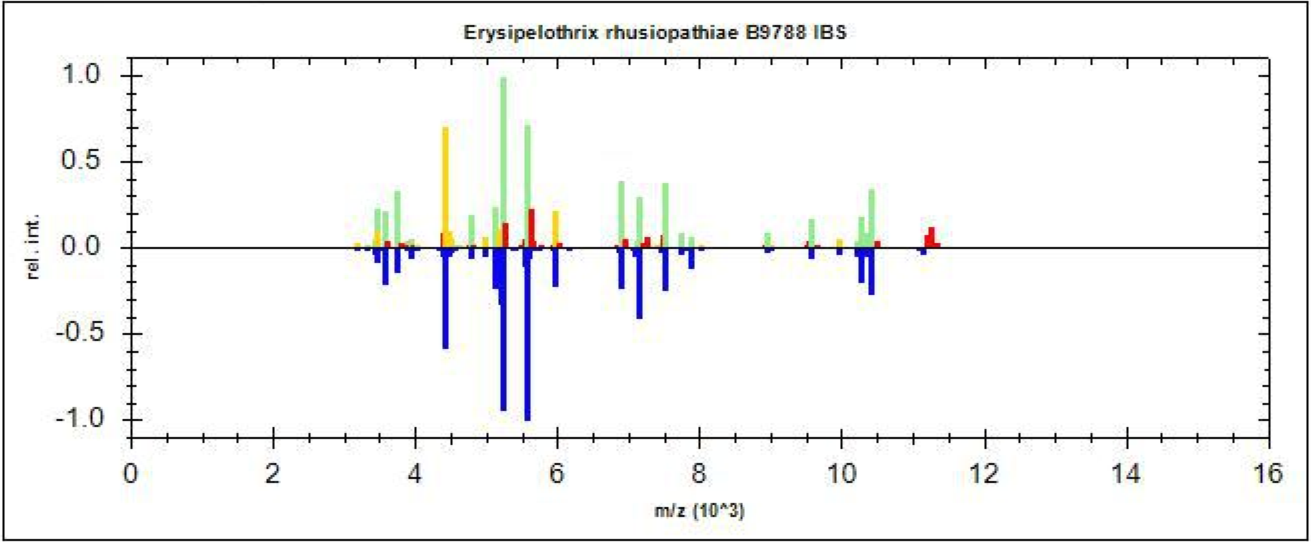

Erysipelothrix rhusiopathiae B9788 IBS
1.0
0.5
0.0
-0.5
-1.0
rel. int.
0
2
4
6
8
10
12
14
16
m/z (10^3)

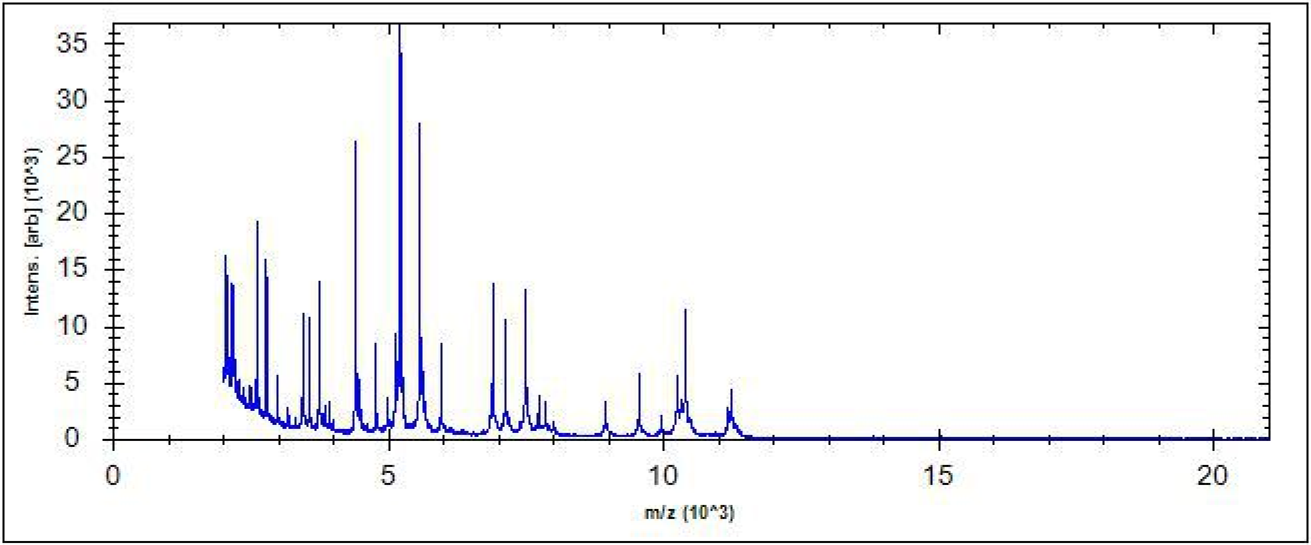

Intens. [arb] (10^3)
35
30
25
20
15
10
5
0
0
5
10
15
20
m/z (10^3)

Bruker MALDI Biotyper Identification Results

BRUKER

Run Info:

Run Identifier:	240923-1329-1011003112
Comment:	
Operator:	tof-user@MBT-WIN10-LTSC
Run Creation Date/Time:	2024-09-23T13:32:01.781
Number of Tests:	3
Type:	Standard
BTS-QC:	not present
BTS-QC Position:	
Instrument ID:	269944.00834
Server Version:	4.1.100 (PYTH) 188 2020-04-112_10-35-53

Result Overview

Sample Name	Sample ID	Organism (best match)	Score Value	Organism (second-best match)	Score Value
B11 (+++) (A)	10110031122409234+#1 (standard)	Escherichia coli	2.21	Escherichia coli	2.19
C12 (+++) (A)	498574#11 (standard)	Erysipelothrix rhusiopathiae	2.17	Erysipelothrix rhusiopathiae	1.95
D1 (+++) (A)	498574#12 (standard)	Erysipelothrix rhusiopathiae	2.27	Erysipelothrix rhusiopathiae	2.14

Matching Hints

Matched Pattern	Comment
Escherichia coli ATCC 25922 CHB	closely related to Shigella / Escherichia fergusonii and not definitely distinguishable at the moment
Escherichia coli ATCC 25922 THL	closely related to Shigella / Escherichia fergusonii and not definitely distinguishable at the moment
Escherichia coli DH5alpha BRL	closely related to Shigella / Escherichia fergusonii and not definitely distinguishable at the moment
Escherichia coli DSM 1103_QC DSM	closely related to Shigella / Escherichia fergusonii and not definitely distinguishable at the moment
Escherichia coli DSM 1576 DSM	closely related to Shigella / Escherichia fergusonii and not definitely distinguishable at the moment
Escherichia coli DSM 682 DSM	closely related to Shigella / Escherichia fergusonii and not definitely distinguishable at the moment
Escherichia coli ESBL_EA_RSS_1528T CHB	closely related to Shigella / Escherichia fergusonii and not definitely distinguishable at the moment
Escherichia coli MB11464_1 CHB	closely related to Shigella / Escherichia fergusonii and not definitely distinguishable at the moment
Escherichia coli Nissl VML	closely related to Shigella / Escherichia fergusonii and not definitely distinguishable at the moment
Escherichia coli RV412_A1_2010_06a LBK	closely related to Shigella / Escherichia fergusonii and not definitely distinguishable at the moment

Meaning of Score Values

Range	Interpretation	Symbols	Color
2.00 - 3.00	High-confidence identification	(+++)	green
1.70 - 1.99	Low-confidence identification	(+)	yellow
0.00 - 1.69	No Organism Identification Possible	(-)	red

Meaning of Consistency Categories (A - C)

Category	Interpretation
(A)	**High consistency:** The best match is a high-confidence identification. The second-best match is (1) a high-confidence identification in which the species is identical to the best match, (2) a low-confidence identification in which the species or genus is identical to the best match, or (3) a non-identification.
(B)	**Low consistency:** The requirements for high consistency are not met. The best match is a high- or low-confidence identification. The second-best match is (1) a high- or low-confidence identification in which the genus is identical to the best match or (2) a non-identification.
(C)	**No consistency:** The requirements for high or low consistency are not met.

Sample 1

**Sample Name:**	B11
**Sample Description:**	
**Sample ID:**	10110031122409234+#1
**Sample Creation Date/Time:**	2024-09-23T13:29:05.061
**Sample Type:**	Standard
**Identification Method:**	MALDI Biotyper MSP Identification Standard Method 1.1
**Preprocessing Method:**	MALDI Biotyper Preprocessing Standard Method 1.1
**ACQ Method:**	D:\Methods\flexControlMethods\MBT_FC.par
**AutoXecute Method:**	MBT_AutoX
**Consistency Category (based on 2 best matches):**	A
**Applied MSP Library(ies):**	BDAL / contains 11897 MSPs / ff6e1dd8-c047-4b48-bb64-1b3eae491247 / 2023-12-13T22:58:22.096, SR_BBFV / contains MSPs of the following four species/groups: Brucella melitensis, Burkholderia mallei/pseudomallei, Francisella tularensis, Vibrio cholerae. / cc6b3c5a-6cdb-43bc-a52b-6e76d11fe42f / 2023-12-13T20:48:17.522

Rank (Quality)	Matched Pattern	Score Value	NCBI Identifier
1 (+++)	Escherichia coli MB11464_1 CHB	2.21	562
2 (+++)	Escherichia coli ATCC 25922 THL	2.19	562
3 (+++)	Escherichia coli DH5alpha BRL	2.15	562
4 (+++)	Escherichia coli DSM 682 DSM	2.15	562
5 (+++)	Escherichia coli RV412_A1_2010_06a LBK	2.09	562
6 (+++)	Escherichia coli DSM 1103_QC DSM	2.09	562
7 (+++)	Escherichia coli DSM 1576 DSM	2.08	562
8 (+++)	Escherichia coli ESBL_EA_RSS_1528T CHB	2.03	562
9 (+++)	Escherichia coli ATCC 25922 CHB	2.00	562

Result table for sample 1--continued on next page

Result table for sample 1--continued from previous page			
Rank (Quality)	Matched Pattern	Score Value	NCBI Identifier
10 (+)	Escherichia coli Nissl VML	1.99	562

Sample 2

Sample Name:	C12
Sample Description:	
Sample ID:	498574#11
Sample Creation Date/Time:	2024-09-23T13:29:05.062
Sample Type:	Standard
Identification Method:	MALDI Biotyper MSP Identification Standard Method 1.1
Preprocessing Method:	MALDI Biotyper Preprocessing Standard Method 1.1
ACQ Method:	D:\Methods\flexControlMethods\MBT_FC.par
AutoXecute Method:	MBT_AutoX
Consistency Category (based on 2 best matches):	A
Applied MSP Library(ies):	BDAL / contains 11897 MSPs / ff6e1dd8-c047-4b48-bb64-1b3eae491247 / 2023-12-13T22:58:22.096, SR_BBFV / contains MSPs of the following four species/groups: Brucella melitensis, Burkholderia mallei/pseudomallei, Francisella tularensis, Vibrio cholerae. / cc6b3c5a-6cdb-43bc-a52b-6e76d11fe42f / 2023-12-13T20:48:17.522

Rank (Quality)	Matched Pattern	Score Value	NCBI Identifier
1 (+++)	Erysipelothrix rhusiopathiae B9788 IBS	2.17	1648
2 (+)	Erysipelothrix rhusiopathiae DSM 5057 DSM	1.95	1648
3 (+)	Erysipelothrix rhusiopathiae ERYB19478 IBS	1.90	1648
4 (+)	Erysipelothrix rhusiopathiae DSM 5056 DSM	1.84	1648
5 (+)	Erysipelothrix rhusiopathiae EDQM Serotyp1 FLR	1.83	1648
6 (+)	Erysipelothrix rhusiopathiae EDQM Serotyp2 FLR	1.81	1648
7 (+)	Erysipelothrix rhusiopathiae FF XI SerotypN FLR	1.76	1648
8 (-)	Erysipelothrix rhusiopathiae ERY604 IBS	1.60	1648
9 (-)	Erysipelothrix rhusiopathiae DSM 5055T DSM	1.57	1648

Result table for sample 2--continued on next page

Result table for sample 2--continued from previous page			
Rank (Quality)	Matched Pattern	Score Value	NCBI Identifier
10 (-)	Erysipelothrix rhusiopathiae DSM 5058 DSM	1.54	1648

Sample 3

Sample Name:	D1
Sample Description:	
Sample ID:	498574#12
Sample Creation Date/Time:	2024-09-23T13:29:05.062
Sample Type:	Standard
Identification Method:	MALDI Biotyper MSP Identification Standard Method 1.1
Preprocessing Method:	MALDI Biotyper Preprocessing Standard Method 1.1
ACQ Method:	D:\Methods\flexControlMethods\MBT_FC.par
AutoXecute Method:	MBT_AutoX
Consistency Category (based on 2 best matches):	A
Applied MSP Library(ies):	SR_BBFV / contains MSPs of the following four species/groups: Brucella melitensis, Burkholderia mallei/pseudomallei, Francisella tularensis, Vibrio cholerae. / cc6b3c5a-6cdb-43bc-a52b-6e76d11fe42f / 2023-12-13T20:48:17.522, BDAL / contains 11897 MSPs / ff6e1dd8-c047-4b48-bb64-1b3eae491247 / 2023-12-13T22:58:22.096

Rank (Quality)	Matched Pattern	Score Value	NCBI Identifier
1 (+++)	Erysipelothrix rhusiopathiae B9788 IBS	2.27	1648
2 (+++)	Erysipelothrix rhusiopathiae ERYB19478 IBS	2.14	1648
3 (+++)	Erysipelothrix rhusiopathiae DSM 5057 DSM	2.12	1648
4 (+++)	Erysipelothrix rhusiopathiae ERY604 IBS	2.02	1648
5 (+++)	Erysipelothrix rhusiopathiae DSM 5056 DSM	2.01	1648
6 (+)	Erysipelothrix rhusiopathiae EDQM Serotyp2 FLR	1.97	1648
7 (+)	Erysipelothrix rhusiopathiae EDQM Serotyp1 FLR	1.93	1648
8 (+)	Erysipelothrix rhusiopathiae DSM 5058 DSM	1.87	1648
9 (+)	Erysipelothrix rhusiopathiae FF XI SerotypN FLR	1.81	1648

Result table for sample 3--continued on next page

Result table for sample 3--continued from previous page			
Rank (Quality)	Matched Pattern	Score Value	NCBI Identifier
10 (+)	Erysipelothrix rhusiopathiae DSM 5055T DSM	1.75	1648
