# Supplementary material for: Erysipelothrix rhusiopathiae-associated bloodstream infection in a patient with systemic lupus erythematosus: a case report and literature review
Source: Access Microbiol. 2024 Nov 6;6(11):000881.v3. doi: 10.1099/acmi.0.000881.v3 (PMC11649197; doi:10.1099/acmi.0.000881.v3)
Supplement: Uncited Supplementary Material 3. [file acmi-6-00881-s003.pdf]

Antimicrobial MIC Susceptibility Tests QC sheet: *S. pneumoniae* (Etest)

Control Organism: ***Streptococcus pneumoniae* ATCC 49619**
Type: Etest strips; MIC in µg/mL
Medium: Mueller-Hinton Agar with 5% Sheep Blood

Perform: weekly (WK) / New shipment (NS)

MONTH/YEAR		2023							
WK / NS		WK		WK		WK		WK + NSL	
DATE				Oct 31		Nov 8		Nov 15	
ANTIBIOTIC & MIC	CODE	Result	Pass / Fail	Result	Pass / Fail	Result	Pass / Fail	Result	Pass / Fail
Amoxicillin 0.03 – 0.12	AC	0.047	P	0.047	P	0.047	P	0.047	P
Ceftriaxone 0.03 - 0.12	TX	0.094	P	0.064	P	0.064	P	0.064	P
Penicillin (Benzylpenicillin) 0.25 - 1.0	PG	0.38	P	0.19	P	0.125	P	0.38	P
Vancomycin 0.12 - 0.5	VA	0.50	P	0.25	P	0.25	P	0.25	P
Initials		RL		GB Nov 1		[illegible]		JS	

Notes:

- **CLSI interpretations are mostly up to 2 decimal places, but strips allow reading up to 3 decimal places.**
- Lot #s and expiry date of media, antibiotic discs and Etest documented on separate spreadsheet.

Technical Coordinator (TC) initials:_______
Technical Practice Lead (TPL) initials:______

Note: This is a controlled document for FHA internal use only. Any documents appearing in paper form are not controlled and should be checked against the signed copy filed with the Laboratory Scientist.

Antimicrobial MIC Susceptibility Tests QC sheet: *S. pneumoniae* (Etest)

Control Organism: *Streptococcus pneumoniae* ATCC 49619
Type: Etest strips; MIC in µg/mL
Medium: Mueller-Hinton Agar with 5% Sheep Blood

Perform: weekly (WK) / New shipment (NS)

MONTH/YEAR		2023							
WK / NS		Wk		WK		Wk		W/K	
DATE		Sep 27		OCT 4		OCT 11		Oct 18	
ANTIBIOTIC & MIC	CODE	Result	Pass / Fail	Result	Pass / Fail	Result	Pass / Fail	Result	Pass / Fail
Amoxicillin 0.03 – 0.12	AC	0.047	P	0.047	P	0.064	P	0.047	P
Ceftriaxone 0.03 - 0.12	TX	0.064	P	0.064	P	0.064	P	0.064	P
Penicillin (Benzylpenicillin) 0.25 - 1.0	PG	0.38	P	0.5	P	0.38	P	0.38	P
Vancomycin 0.12 - 0.5	VA	0.38	P	0.5	P	0.25	P	0.5	P
Initials		Lz		SS		Lz		W	

Notes:

- **CLSI interpretations are mostly up to 2 decimal places, but strips allow reading up to 3 decimal places.**
- Lot #s and expiry date of media, antibiotic discs and Etest documented on separate spreadsheet.

Technical Coordinator (TC) initials:_______
Technical Practice Lead (TPL) initials:______

Note: This is a controlled document for FHA internal use only. Any documents appearing in paper form are not controlled and should be checked against the signed copy filed with the Laboratory Scientist.
